# SARS-CoV-2 spread and area economic disadvantage in the italian three-tier restrictions: a multilevel approach

**DOI:** 10.1186/s12889-023-15246-1

**Published:** 2023-02-14

**Authors:** Luca Dei Bardi, Anna Acampora, Laura Cacciani, Mirko Di Martino, Nera Agabiti, Marina Davoli, Giulia Cesaroni

**Affiliations:** 1 Department of Epidemiology - Regional Health Service, ASL Roma 1, Rome, Italy; 2 Sapienza University of Rome, Rome, Italy

**Keywords:** SARS-CoV-2, Tiered restrictions, Socioeconomic, Multilevel analysis, Non-pharmacological intervention

## Abstract

**Background:**

To face the second wave of COVID-19, Italy implemented a tiered restriction system with different limitation levels (yellow = medium; orange = medium-high, red = high) at the beginning of November 2020. The restrictions systematically reduced the transmission of SARS-CoV-2 with increasing strength for increasing tier. However, it is unknown whether the effect of limitations was equal between provinces with different socioeconomic levels. Therefore, we investigated the association between the province’s socioeconomic level and SARS-CoV-2 infection daily reproduction number in each restriction level.

**Methods:**

We measured the province’s socioeconomic level as the percentage of individuals whose 2019 total yearly income was lower than 10,000€, using the measure as a proxy of economic disadvantage. We estimated the daily reproduction number (Rt) at the province level using the SARS-CoV-2 daily incidence data from November 2020 to May 2021. We then used multilevel linear regression models with random intercepts stratified by restriction level to estimate the association between economic disadvantage and Rt. We also adjusted the analyses for potential confounders of the association between the province’s economic disadvantage and the Rt: the percentage of people with 0–5 years, the quartiles of population density, and the geographical repartition.

**Results:**

Overall, we found increasing Rt in yellow (+ 0.004 p < 0.01, from Rt = 0.99 to 1.08 in three weeks) and containing effects for the orange (-0.005 p < 0.01, from Rt = 1.03 to 0.93) and the red tier (-0.014 p < 0.01, from Rt = 1.05 to 0.76). More economically disadvantaged provinces had higher Rt levels in every tier, although non-significantly in the yellow level (yellow = 0.001 p = 0.19; orange = 0.002 p = 0.02; red = 0.004 p < 0.01). The results showed that the association between economic disadvantage and Rt differed by level of restriction. The number of days into the restriction and the economic disadvantage had statistically significant interactions in every adjusted model. Compared to better off, more economically disadvantaged provinces had slower increasing trends in yellow and steeper Rt reductions in orange, but they showed slower Rt reductions in the highest tier.

**Conclusion:**

Lower tiers were more effective in more economically disadvantaged provinces, while the highest restriction level had milder effects. These results underline the importance of accounting for socioeconomic level when implementing public health measures.

**Supplementary Information:**

The online version contains supplementary material available at 10.1186/s12889-023-15246-1.

## Background

It is well known that socioeconomic level is associated with health outcomes [1, 2]. The SARS-CoV-2 pandemic is no exception, as it exacerbated pre-existent socioeconomic inequalities regarding finances and basic needs [3, 4]. Moreover, evidence from the first wave showed associations between socioeconomic level and the spread of the virus in the US [5–7], Europe [8–10], and Italy [11, 12]. Furthermore, lockdown and other measures to prevent the spread of SARS-CoV-2 had a different impact on mobility depending on individual and contextual socioeconomic levels, showing lower compliance to stay-at-home orders by poorer neighborhoods [13–15].

After one of the longest lockdowns worldwide, Italy gradually lifted all restrictions during the summer of 2020, when only an indoor mask mandate was in place [16]. In November 2020, when facing the second wave, Italy implemented a three-tier restriction system based on a pandemic threat assessed at the regional level (second level of the European Nomenclature of Territorial Units for Statistics, NUTS-2). This system defined the pandemic threat and the restrictions combining three levels of risk based on 21 indicators and four transmission levels [17]. The pandemic threat could be either “moderate” (yellow; default level), “elevated” (orange; high risk and medium-high transmission), or “maximum” (red; high risk and transmission), where each level had a set of increasing restrictions and was named according to the color scheme. Specifically, in the yellow tier, the limitations were: mandatory face masks indoors and outdoors, halved public transport capacity, distance learning in high schools and universities, closure of shopping malls on weekends and holidays, closure of some indoor activities like cinemas, exhibitions, museums and gyms, stop to service in bars and restaurants at 18:00, and a curfew between 22:00–5:00. The additional restrictions in the orange tier were the stop to travels between municipalities and regions and the suspension to all non-delivery food-service activities. Last, the red level had a complete lockdown where only essential workers were allowed to move, and only primary schools kept their in-presence everyday activities. In mid-January 2021, the pandemic threat assessment procedure was updated introducing a threshold of 50 weekly cases per 100.000 into the evaluation. This revision allowed the implementation of higher restrictions in regions with high incidence but a medium level of risk or low transmission [18]. In the same period, the system added a new lowest level of restrictions: when regions reached the so-called “low” pandemic threat (white, low risk, low transmission, and low incidence), only a mask mandate indoors was in place. A more in-depth description of all tiers can be found elsewhere [18–21].

This restriction system has been shown to systematically reduce mobility between and within regions when higher tiers were implemented [19, 20]. Moreover, a study on the nine most populated Italian regions showed stronger reductions in transmissibility with increasing tiers [21]. However, it is unknown whether these effects were equal among provinces characterized by different levels of economic disadvantage. It is well known that adherence to stay-at-home orders implemented during the SARS-CoV-2 pandemic differs among socioeconomic groups [13–15, 22, 23]. However, whether this remains true for less strict mandates remains an open question.

Our main goal was to investigate the association between the province’s economic disadvantage and SARS-CoV-2 spread by the level of restriction.

## Methods

### Study design and setting

We conducted an ecological study at the province level, corresponding to the NUTS-3 regions. Due to the timing of the restrictions’ implementation (beginning of November 2020) and a disruptive change in the pandemic threat assessment procedure in mid-May 2021, we analyzed the second pandemic wave. Specifically, the period from Sat 6 Nov 2020 to Sun 9 May 2021.

### Unit of observation and variables

In this study, the units of observation are the 107 Italian provinces. For each province, we measured the exposure (economic disadvantage), the daily outcome (infection spread), the potential confounders (population density, population age structure, and geographical repartition), and the number of days into each restriction tier.

We measured the provinces’ economic disadvantage (PED) using data on total yearly income from the Ministry of Economy and Finance [24]. We calculated the PED using the percentage of taxpayers in 2019 with a total yearly income lower than €10,000. The total yearly income includes all gross incomes from work (employment, self-employment, and pensions) and other sources (capital, land, and business).

To measure the SARS-CoV-2 spread, we used the cumulative number of SARS-CoV-2 infections disseminated daily by the Italian Civil Protection Department [25]. The dataset contains information about the cases confirmed through Reverse Transcription-Polymerase Chain Reaction (RT-PCR). We estimated the SARS-CoV-2 spread through the daily reproduction number (Rt) using Cori’s “instantaneous Rt” methodology [26, 27]. In this setting, Rt is the sum of infection incidences observed in the previous period weighted by the infectivity function. We assumed that the infectivity function followed a Gamma Distribution with parameters estimated in the first outbreak in Lombardy [28].

We observed the provinces’ extensions and population age structure from the National Institute of Statistics [29–31]. We estimated the population density for every province and then we divided the provinces into quartiles. To account for the mask mandate exemption for children, we used the percentage of individuals aged 0–5 (population age structure). Last, to account for the geographical repartition of the provinces, we aggregated the five NUTS-1 classifications in three groups, considering Northeast and Northwest as “Northern” Italy and South and Insular as “Southern” Italy.

The Italian Ministry of Health communicated weekly the change in pandemic threat assessments and restrictions in each region, and we gathered the information from the Ministry of Health’s web page about the novel coronavirus and the SKY TG24 news archive [32, 33]. We did not analyze the “low” level of pandemic threat, namely the white, because only the five Sardinia’s provinces reached the level in the study period.

### Statistical analysis

We used graphical displays for descriptive geographical analysis, timing of restriction by region, and trend of Rt and incidence at the national level. We also plotted Rt trends for each province in the single restriction episode by PED tercile and tier of restriction along with their average trend and 90% credible interval. The average trend is calculated through multilevel linear regression models stratified by restrictions and economic disadvantage terciles with days as the only covariate. The 90% credible intervals are obtained through simulation of the posterior distribution of the day parameters [34].

We used Multilevel Linear Regression (MLR) models with random intercepts stratified by restriction tier to analyze the association between PED and Rt. That is, per every restriction we defined Rt as: ${Rt}_{ij}= \alpha +{{x}}_{ ij}^{{\prime }}\beta +{u}_{i}+{e}_{ij}$ where $i$ indicates the cluster and $j$ the observation. In the formula, $\alpha$ is the fixed intercept, ${{x}}_{ ij}^{{\prime }}$ is the vector of variables observed in cluster $i$ at the $j$-th observation, $\beta$ the vector of fixed effects (common for every cluster and observation), ${u}_{i}$ the specific random intercept of cluster $i$ assumed to be normally distributed, and ${e}_{ij}$ the usual error term of linear regressions assumed to be normally distributed as well. Since provinces faced the same restriction several times in different pandemic contexts, we choose to not define the cluster $i$ of MLR models as the province. Rather, we preferred to define a finer cluster $i$ as the province in the single episode of restriction. Hence, every province in each color limitation has different intercepts every time it faces the restriction. To check whether MLR models were needed, we estimated the Intraclass Correlation Coefficient (ICC) for every restriction level. We ran four sets of MLR models. First, we ran days models to estimate the overall effect of each tier. The estimates for the number of days into the restriction allowed us to identify the average daily effect of the restriction itself. Second, we ran PED models to study the average association of PED with our response variable. Third, we ran MLR models with the linear effect of PED, the linear effect of days, and their interaction. In this setting, studying the interaction term gives insights into the differential effect of PED by tier. While linear effects represent changes in the Rt starting level, the interaction term represents a change in the Rt trend, that is, the slope. Fourth, we added all the confounders to the models with interaction, obtaining adjusted models. All the model equations are reported in the supplementary material. For all models, we reported estimates, standard errors, and p-values, considering estimates with p < 0.05 as statistically significant. Moreover, to ease the interpretation of interaction terms in the models, we calculated the turning points for both PED and days. Turning points are the values at which the first-order partial derivative of the function equals zero. As in typical function studies, it is possible to know whether the outcome variable increases or decreases with larger or smaller values than the turning point.

Finally, we ran two sensitivity analyses. First, we removed restrictions shorter than a week and ran MLR full models to check whether short restrictions can affect the results. Then, we ran MLR full models weighted by the provinces’ populations, giving more weight to more populated provinces which in turn have more stable and reliable Rt.

In the analyses, we centered the economic disadvantage and the share of people aged 0–5 to their minimum observed values (19.8 and 3.4 respectively). This allows the intercepts to have a theoretically observable value in all models. We used the software R for data preparation and analyses [35]. All data and codes are available in an online repository to guarantee full access and reproducibility to our results [36].

**Fig. 1:**
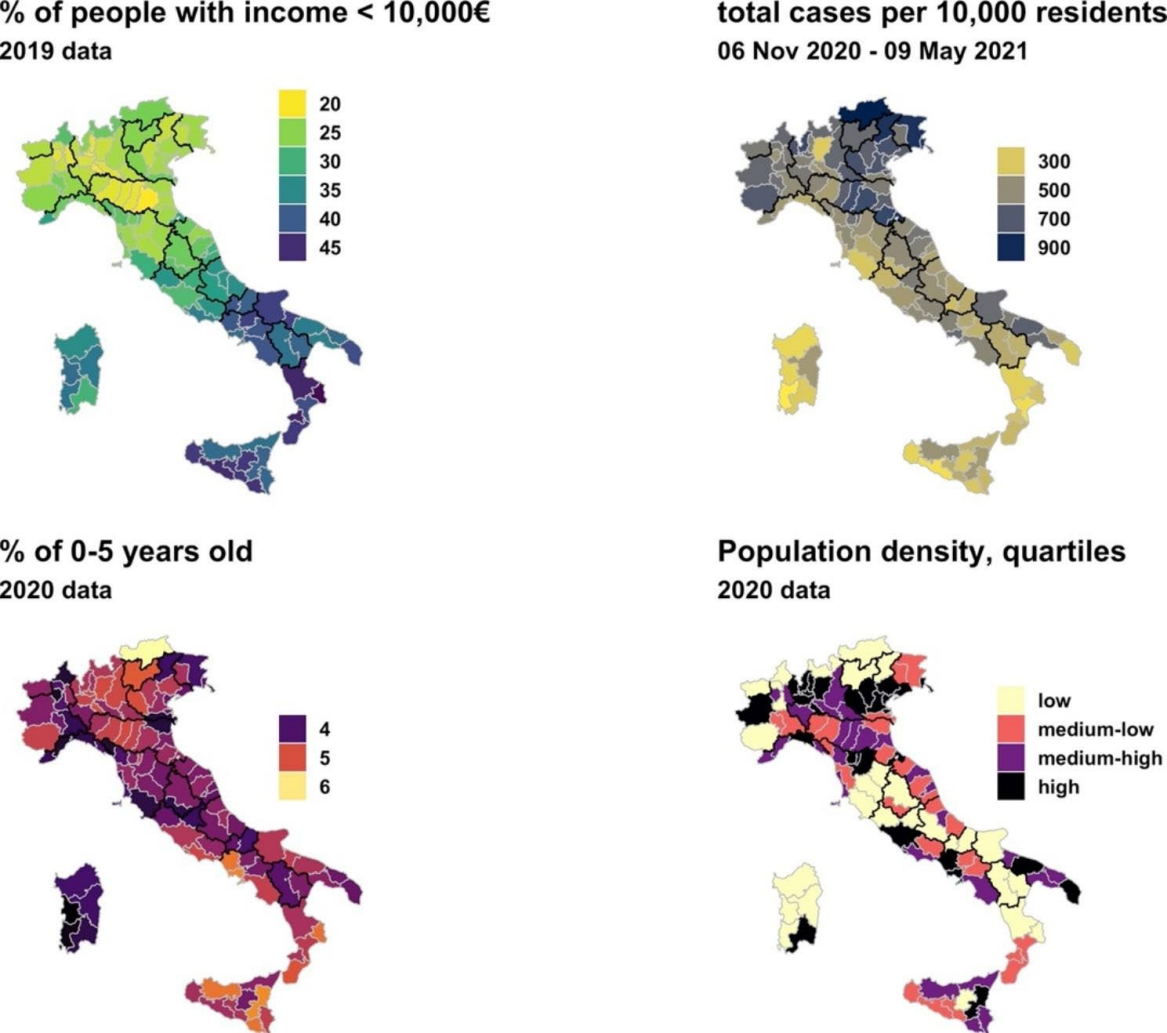
Selected variables by Italian provinces (NUTS-3 level)

## Results

In Italy, there are 107 provinces (NUTS-3 level) heterogeneous by extension, population, and income distribution. These three variables varied between 203 and 7,692 square kilometers, 83 − 4,253 thousand people, and 19.8–48.5% of people with low income (PED), respectively.

Figure 1 shows the Italian maps by provinces of the variables we considered: the percentage of people with a gross yearly income lower than €10,000; the SARS-CoV-2 cases per 10,000 inhabitants in the period 06 Nov 2020–09 May 2021; the percentage of people aged 0–5 years; and the population density quartiles. In the figure, a remarkable north-south gradient in yearly income is visible. Southern provinces consistently show a larger share of low-income people than northern provinces. However, the reverse is observed for SARS-CoV-2 infections per 10,000 inhabitants. Higher values in cumulative incidence of cases were observed in the north, suggesting an inverse relationship between our measure of PED and the spread of SARS-CoV-2 infection. The share of the population aged 0–5 is generally higher in the north and south of Italy, with lower values observed in Sardinia and the center. Finally, population density is the lowest in mountainous provinces and the highest in provinces with the largest cities in Italy.

**Figure S1**, in the supplementary material, reports the regional daily measures in place from 1 to 2020 to 17 May 2021, highlighting the date when the pandemic threat assessment started including the weekly incidence threshold (16 January 2021). Restrictions changed frequently and were heterogeneous by duration and implementation, showing no association with the geographical repartition of the regions. Country-wide restrictions not based on the pandemic threat of the regions are visible during Christmas, new year, and Easter (4 Apr 2021) periods. **Figure S2**, which can be found in the supplementary material, displays the trends of Rt and SARS-CoV-2 incidence from the beginning of data collection on positives in Italy (24 Feb 2020) to 31 Dec 2021, highlighting the study period. Confronting **Figure S1** and **Figure S2** is visible that the Italian Government implemented the first restrictions right before November’s pandemic peak. Also, a new peak occurred in mid-March, this led many regions to the orange or the red restrictions before the so-called “Easter lockdown” was imposed at the national level.

**Fig. 2:**
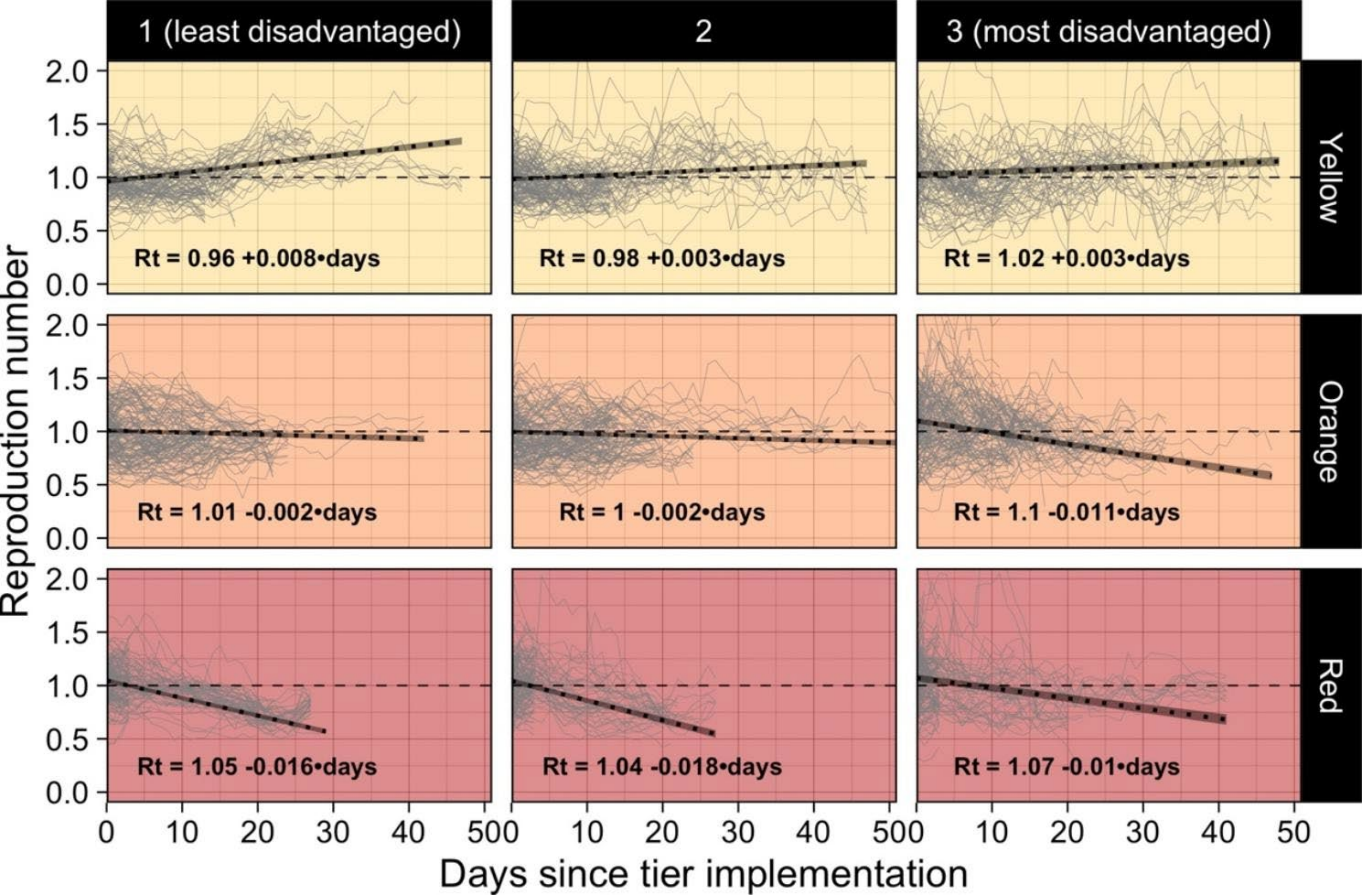
Reproduction number trends by economic disadvantage terciles and restriction levels

Figure 2 shows the Rt trends observed from the restriction implementation by PED terciles and restriction levels. Overall, the yellow tier showed increases in Rt, while the orange and the red reduced the spreading of the virus with greater reductions in the red compared to the orange tier. However, we can observe very different slopes between the least and the most economically disadvantaged in every level of restriction. While the overall trend in the yellow tier was rapidly increasing for the least disadvantaged provinces, the other two terciles showed slower growths. Also, the most economically disadvantaged appeared to have the sharpest decreasing slope in the orange tier. The opposite is visible in the highest restriction level, where the reduction in Rt is less steep in provinces with the highest share of people with low income.

Table 1 shows the results from MLR models with fixed effects and random intercepts stratified by level of restriction. To account for the strong correlation of subsequent Rt, we defined the cluster as the single episode in which the province entered a tier. Estimates of the Intraclass Correlation Coefficient (ICC) reported in the table confirm the need for a multilevel analysis as clusters explained 42–63% of the Rt variability (ICC yellow = 0.41; ICC orange = 0.63; ICC red = 0.55). Results from days models (Table 1.**A**) showed an increasing Rt trend for the yellow tier (days = 0.004 p < 0.01), a small restrictive effect in the orange tier (days = -0.005 p < 0.01), and a strong effect in the red tier, where every day of restriction contributed to reducing the Rt by 0.014 (p < 0.01). On average, Rt increased from 0.99 to 1.08 after three weeks in the yellow level, decreased from 1.03 to 0.93 in the orange restriction, and reduced from 1.05 to 0.76 in the red tier. Table 1.**B** shows that provinces with higher PED have always higher Rt in every restriction level, although non-significant in the lowest tier (yellow = 0.001 p = 0.19; orange = 0.002 p = 0.02; red = 0.004 p < 0.01). Interaction MLR models in Table 1.**C** confirmed the trends observed in Fig. 2. The yellow tier model shows a positive effect of days and PED and a negative statistically significant interaction term, representing a slower increase in Rt for more economically disadvantaged provinces. The positive effects of both PED and days were reversed at the turning points PED = 64.7, outside the observed range, and days = 16.4. These estimates and the negative sign of the interaction effect show an increasing daily trend of Rt for all PED levels and a lower Rt for higher PED after 17 days in the yellow tier. The interaction model of the orange level reports a significant change in the direction of the effect of days. However, this inversion is completely balanced by the statistically significant interaction effect of days and PED. The negative estimate for the interaction term reverses the two positive estimates for the single terms at PED = 21.9 and days = 10.4. Given that the minimum observed share of low-income people is 19.8, a few provinces had a slightly increasing trend while every other province had a reduction in the spread of the virus when in the orange tier. The reduction in Rt had growing strength with greater PED. On average, after 11 days into the orange level, the starting gap between more and less economically disadvantaged provinces was filled and reversed.

**Table 1:** Association between economic disadvantage and SARS-CoV-2 spread by restriction tier

	Yellow (ICC = 0.418)			Orange (ICC = 0.632)			Red (ICC = 0.553)		
	Est.	SE	p	Est.	SE	p	Est.	SE	p
**A) days models**									
Intercept	0.989	0.008	< 0.01	1.032	0.008	< 0.01	1.050	0.009	< 0.01
days	0.004	2E-04	< 0.01	− 0.005	2E-04	< 0.01	− 0.014	3E-04	< 0.01
**B) PED models**									
Intercept	1.008	0.013	< 0.01	0.981	0.013	< 0.01	0.942	0.015	< 0.01
PED	0.001	0.001	0.19	0.002	0.001	0.02	0.004	0.001	< 0.01
**C) interaction models**									
Intercept	0.967	0.014	< 0.01	0.973	0.013	< 0.01	1.026	0.015	< 0.01
days	0.006	4E-04	< 0.01	0.001	4E-04	< 0.01	− 0.018	0.001	< 0.01
PED	0.002	0.001	0.07	0.006	0.001	< 0.01	0.002	0.001	< 0.01
PED x days	-1E-04	3E-05	< 0.01	-5E-04	3E-05	< 0.01	3E-04	4E-05	< 0.01
D) adjusted* models									
Intercept	0.928	0.028	< 0.01	0.985	0.026	< 0.01	0.993	0.030	< 0.01
days	0.006	4E-04	< 0.01	0.001	4E-04	< 0.01	− 0.018	0.001	< 0.01
PED	-0.002	0.003	0.55	0.005	0.002	0.05	0.009	0.003	< 0.01
PED x days	-1E-04	3E-05	< 0.01	-5E-04	3E-05	< 0.01	3E-04	4E-05	< 0.01

Estimates from Multilevel Linear Model with random intercepts stratified by restriction tier. ICC = Intraclass Correlation Coefficient; Est. = Estimate; SE = Standard Error; PED = Province’s Economic Disadvantage. *Models were adjusted for population density, share of people aged 0–5, and geographical repartition

Finally, the interaction model of the red tier confirmed the slower reductions in Rt for more economically disadvantaged provinces observed previously. The negative effect of days on Rt was eased by the positive effect of both PED and the interaction term. Turning points happened to be outside the observed range of the variables (PED = 71.8, days = -7.2), meaning that provinces with a higher share of people with low income always had both higher Rt levels and slower reductions in the red tier. When adjusting the estimates for potential confounders, we observed little to no differences in our variables of interest (Table 1.**D**). The estimate of PED in the yellow tier model became negative but remained non-significant causing a sign change in the turning point of days as well (days = -11.6). The adjusted model for the yellow tier thus shows that provinces with a higher share of low incomes always had lower starting Rt (although non-significant) and lower increasing trends, thus resulting constantly advantaged in the lowest tier. The orange level adjusted model showed no meaningful difference with its non-adjusted peer. Last, the red tier adjusted model differed in the effect size of PED only (PED = 0.009; p < 0.01), but do not differ in meaning from the non-adjusted.

The confounding variables did not have statistically significant effects (not shown). It is worth underlining two exceptions: (1) the effect of the share of children aged 0–5 in the yellow tier model (estimate = 0.054 p < 0.01), and (2) the significant effect of NUTS-1 regions in the red tier model (Central vs. Northern: -0.078 p < 0.01; Southern vs. Northern: -0.138 p < 0.01) that numerically represent what showed in Fig. 1.

Sensitivity analyses performed on adjusted models are reported in **Table S1** and showed no substantial change in the reported results. Models without restrictions in place for less than 7 days, shown in **Table S1.1** of the supplementary material, did not have different estimate directions or significance of the adjusted models. Also, weighting the adjusted models for the population of the provinces did not change our estimates, as reported in **Table S1.2** in the supplementary material.

## Discussion

In our analysis of Italian provinces, we found differential effects of the provinces’ economic disadvantage on SARS-CoV-2 spread by restriction level. We found statistically significant interaction effects between the number of days into the restriction and the PED. Overall, in the lowest tier (yellow), Rt had an increasing trend, but more economically disadvantaged provinces had slower Rt increases than less disadvantaged. This different behavior resulted in generally higher Rt values for the latter. Conversely, a moderate level of restrictions (orange) led to a general decrease in Rt values. The more economically disadvantaged provinces resulted particularly benefited from the orange tier, as their Rt downward trend was steeper than less disadvantaged provinces. The average level of Rt was lower in more disadvantaged than less disadvantaged provinces after 9 days in the orange level. However, in the highest level of restriction (red) the higher the PED, the higher the Rt, and the slower the reduction.

Our results suggest the presence of different behaviors in the three levels of restrictions depending on the PED. The yellow level of restrictions was mainly characterized by the nocturnal curfew, mandatory stop to services in bars and restaurants at 18:00, and closure of social indoor activities (i.e., cinemas, gyms, museums) and shopping malls on non-working days. However, many social activities were still allowed during the day. When these restrictions were in place, we could expect that people with a wider economic availability enjoyed more frequently the restaurants, bars, shopping centers, and all the other available social venues. In contrast, disadvantaged people could spontaneously stay more at home, resulting in lower transmissibility. This could result in a higher number of social contacts and higher chances of contagion in less economically disadvantaged provinces compared to more disadvantaged ones and might explicate the faster increasing Rt trends for the least disadvantaged provinces when the lowest level of restrictions was applied.

The orange tier, in addition to the restrictions applied in yellow, introduced a suspension to most social activities and limited the mobility between municipalities and regions. However, those limitations did not demonstrate a higher impact in less compared to more economically disadvantaged provinces. A possible explanation of the observed trend for less disadvantaged could be that in the orange tier, although stricter, the measures did not introduce a stay-at-home order, did not forbid private gatherings, and did not mandate smart working. That is, “non-essential” in-person working activities could continue. Moreover, it is plausible that the restricted mobility between municipalities impacted more residents in small towns than those living in big cities, and provinces with large provincial capitals have lower percentages of people with low income than those composed of small conurbations. However, albeit it is safe to attribute the reduced spread of SARS-CoV-2 to the reduced mobility between municipalities and the take-away-only mandate to restaurants and bars, the reasons why these measures resulted more effective in more economically disadvantaged provinces remain an open question.

The highest level of restrictions (red) corresponds to a complete lockdown, where only essential workers were allowed to leave home. In this case, we could speculate that more economically disadvantaged provinces have higher shares of essential low-skilled workers who do not have the chance to work at home whilst limiting contacts and rapidly reducing the Rt [15, 37]. In addition, essential low-skilled workers could also have limited access to personal protective equipment and safe working conditions are generally less guaranteed [38].

In summary, while more advantaged people would have more contacts with lower restrictions, those with less economic availability would have more unavoidable social contacts during lockdowns increasing the chance of infections occurring.

Our results about the red tier are comparable to previous studies about the effect of lockdowns during the first wave. For example, several studies found lower efficacy of lockdowns in poorer neighborhoods or disadvantaged areas in the United States [5–7], Europe [8–10], and Italy [11, 12]. Furthermore, our results are coherent with a European Commission report on the impact of the Italian three-tier system on mobility, which found lower mobility with increasing restriction levels [20]. Also, our findings on the restrictions’ effects are consistent with previous studies that found increasing Rt trends with the yellow restrictions, little to no reduction in the orange tier, and sharp declines in Rt with the red level [19, 21].

This work has its limitations. First, it was an ecological study, and ecological fallacy can lead to associations not necessarily true at the individual level. Although we used the lowest-available data reaching the NUTS-3 level, the granularity remains high, and it is not possible to account for individual confounders. Second, the restrictions were decided based on the assessed pandemic threats at the regional level (NUTS-2) while our analyses were at a lower level (NUTS-3). This means that provinces of the same region with different spread and control of the virus could face the same restriction, potentially modifying the strength of our estimates unpredictably. Third, our measure of PED based on the gross yearly income did not consider the difference in the cost of living, the unofficial labor, and other forms of socioeconomic disadvantage. The same yearly income could have very different purchasing power in different parts of Italy. In fact, a report by the Bank of Italy estimated that the cost of living is roughly 17% lower in south-insular than in north-central Italy[39]. Unofficial labor is also more widespread in southern Italy. Both phenomena could affect our measure of PED by showing more people with low income than the actual value. However, a study found that price differentials do not compensate for the differences in incomes, although they reduced the north-south gradient [40]. Regardless of all, the threshold we used of € 10,000 per year is very low, and gross incomes lower than this threshold are hardly considered adequate for living in any part of Italy. Moreover, informal work could also be intended as a form of disadvantage for its lack of economic security and absence of sick leave. Fourth, during the study period, the vaccination campaign began (27/12/2021), and the new alpha variant (lineage B.1.1.7) became predominant, replacing the original SARS-CoV-2 strain. We choose to not account for those two variables in our analyses because it is easy to assume that the share of vaccinated people is a mediator of the PED on SARS-CoV-2 spread, thus, adjusting our analyses for the vaccinal status of the population would remove part of the total effect we are interested in. Also, to the best of our knowledge, the alpha variant was associated with higher spread, but there is no evidence that it was associated with the economic disadvantage of the province, making it not a confounder between the two measures. Moreover, public data on the vaccinal status of the population and the spread of the alpha variant are only available at the regional level [41, 42]. Fifth, the data on SARS-CoV-2 infections might have some delay in the notification that is hardly quantifiable and may be affected by diverse under-notification by province. The former could assign infections that occurred previously to a sequent tier, affecting the starting and ending Rt we observed in each restriction and province. Regarding the latter, it is possible that smaller or more economically disadvantaged provinces were more affected by the under-notification of cases due to lower detection capability. This would result in lower reported infections during outbreak peaks and underestimations of Rt. Sixth and last, we assumed linear trends for Rt in every tier. This does not account for a plateau effect of the tiers or pandemic fatigue by the population. However, as visible in Fig. 2, implementation periods were relatively short and trends, on average, appear as linear.

Our work also has some strengths. First, to our knowledge, this is the first study to look at inequalities in the effects of tiered restrictions. It underlines differential behaviors from areas characterized by different incomes, analyzing the effect on the spread of SARS-CoV-2. Second, our study is based on a reliable measure of Rt, widely used in the literature and the daily reports of the Italian National Institute of Health [43–45]. The instantaneous Rt does not need any assumption except the distribution of the infectivity function and, intrinsically, that the observed past trend will hold in the close future. Mainly, the use of this measure does not require any assumption about the growth of the epidemic, often assumed to be exponential, which is rarely met in context with changing restricting measures such as the one we analyzed. Finally, our estimates were robust to sensitivity analyses either with removed shorter periods of restrictions or weighted by provinces’ population. To ease the replicability to the readers, the full code and data can be found in an online repository [36].

Our results suggest the importance of improving public policies at area levels that make it possible to account for the composition of the population. Consequently, resources could be allocated based on evaluated needs. In more economically disadvantaged areas, these policies could pay particular attention to workers in essential services that cannot work at home by implementing, for example, specific preventive measures aimed to limit virus circulation within the workplace [38, 46]. As suggested by some authors, workers with symptoms or known contact with a positive person should be encouraged to stay at home without the risk to lose their job, and free onsite testing could be offered also facilitating access to diagnosis [38]. Also, in less economically disadvantaged areas more efforts could be oriented to strengthen the opportunity to work at home and to implement education and information campaigns in the context of social activities.

## Conclusion

This study found that the associations between area-level economic disadvantage and the spread of SARS-CoV-2 differed for diverse levels of restriction implemented to prevent the spread of the virus. While lower restrictions curbed more the spread in more economically disadvantaged provinces, the lockdown reduced more the spread in less disadvantaged provinces. We hypothesize that these results could be linked, at least in part, to different shares of people with low income and essential workers. This study suggests the importance of further differentiating actions, aiming at both minimizing the burden on the population and maximizing the impact of the restrictions on the spread of epidemics/pandemics. This would allow for early ease or early implementation of restrictions, as well as aimed policies shaped for specific contexts, optimizing the outcomes. This work calls for new studies to investigate whether associations found at the province level are also present at the municipal or individual level.

## Electronic supplementary material

Below is the link to the electronic supplementary material.

Supplementary Material 1

Supplementary Material 2

Supplementary Material 3

## Data Availability

The datasets generated and/or analyzed during the current study are available in a GitHub repository, https://github.com/Luca-DB/covid_provincia.

## List of abbreviations

ICC: Intraclass Correlation Coefficient
MLR: Multilevel Linear Regression
NUTS: Nomenclature of Territorial Units for Statistics
PED: Provinces’ Economic Disadvantage
Rt: Reproduction number
