# Supplementary figures and images for: SARS-CoV-2 spread and area economic disadvantage in the italian three-tier restrictions: a multilevel approach
Source: BMC Public Health. 2023 Feb 14;23:329. doi: 10.1186/s12889-023-15246-1 (PMC9926448; doi:10.1186/s12889-023-15246-1)

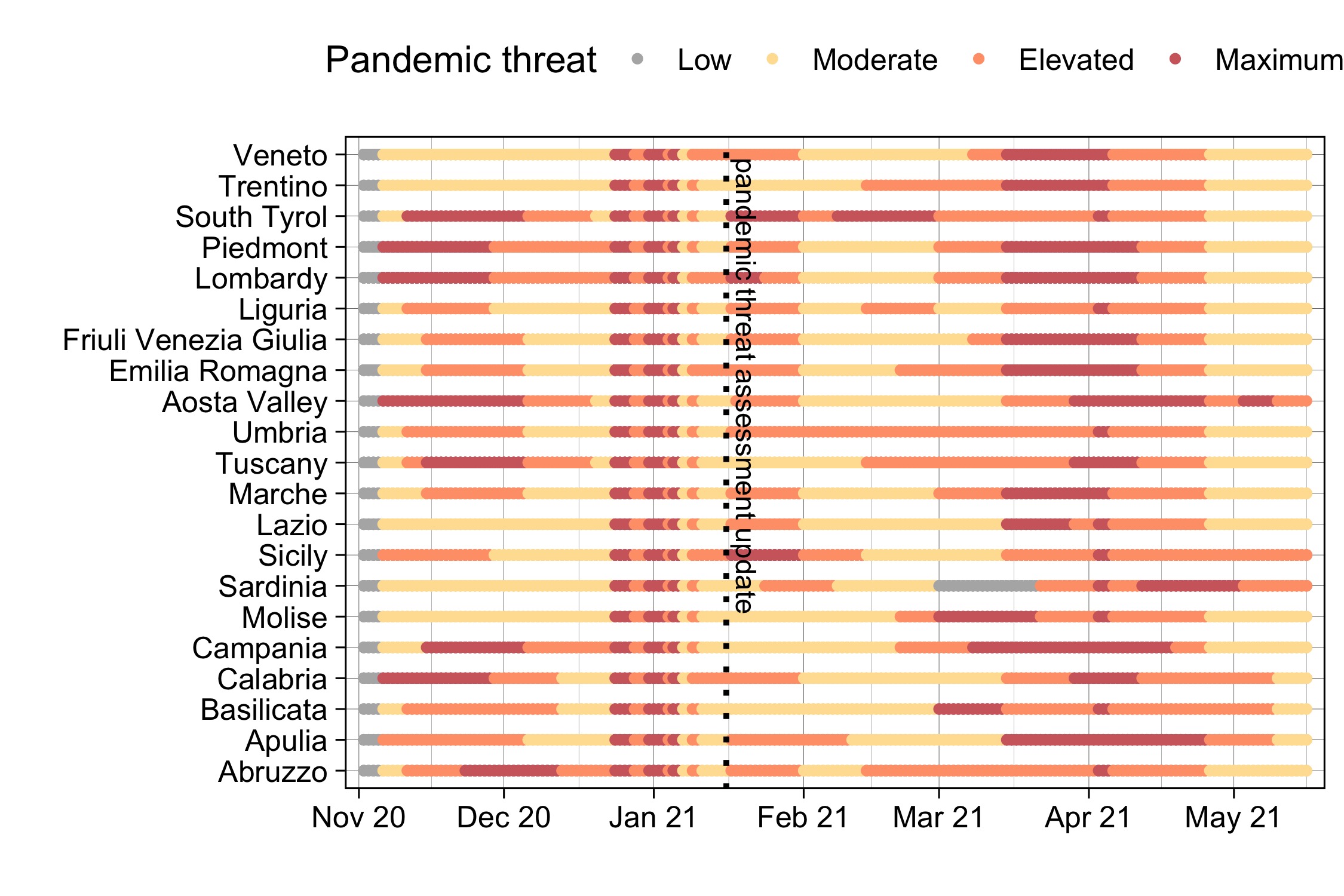

Supplement: Supplementary file 1 — Supplementary Material 1 [file 12889_2023_15246_MOESM1_ESM.jpg]

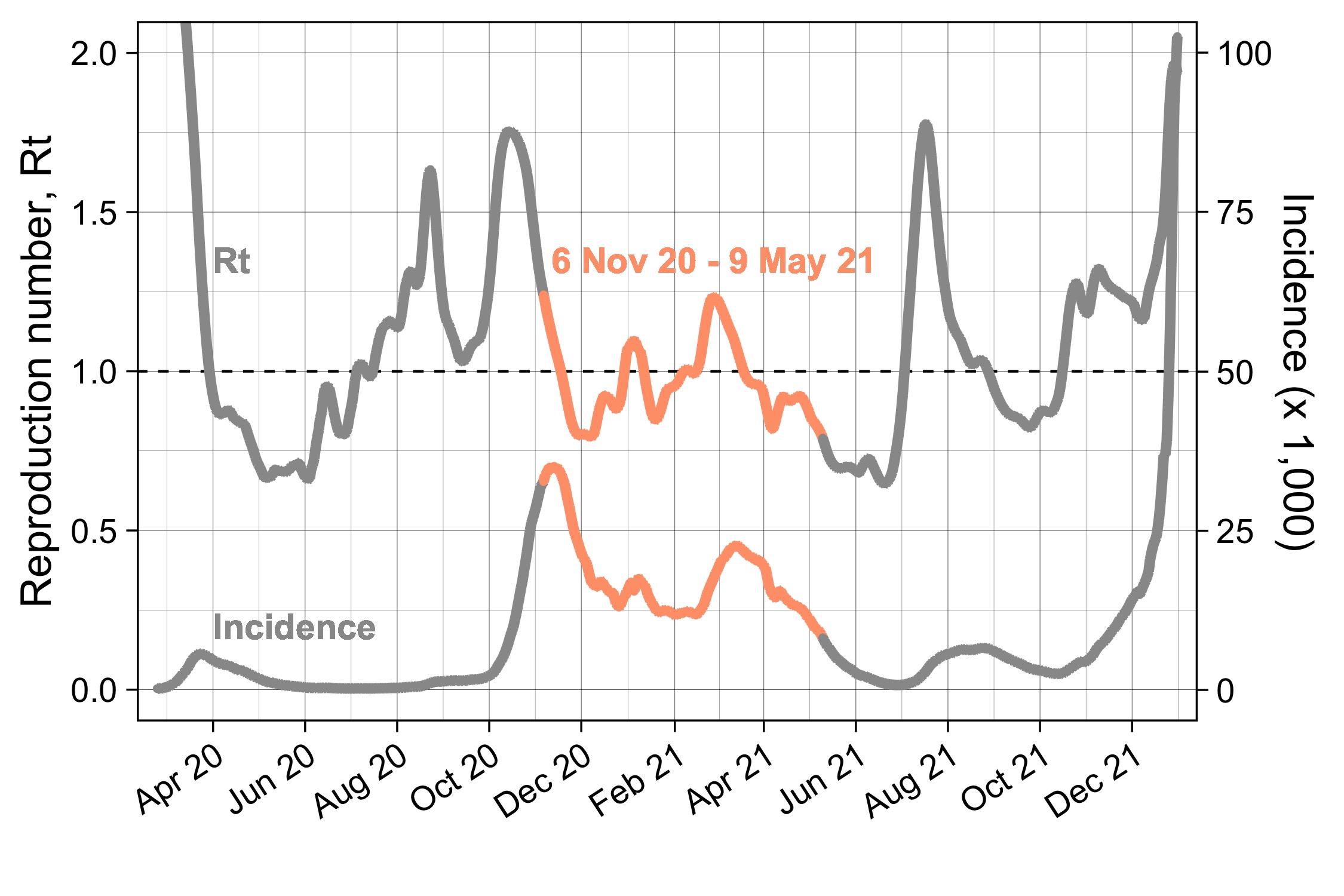

Supplement: Supplementary file 2 — Supplementary Material 2 [file 12889_2023_15246_MOESM2_ESM.jpg]
