# Supplementary material for: SARS-CoV-2 spread and area economic disadvantage in the italian three-tier restrictions: a multilevel approach
Source: BMC Public Health. 2023 Feb 14;23:329. doi: 10.1186/s12889-023-15246-1 (PMC9926448; doi:10.1186/s12889-023-15246-1)
Supplement: Supplementary file 3 — Supplementary Material 3 [file 12889_2023_15246_MOESM3_ESM.docx]

## Title

SARS-CoV-2 spread and area economic disadvantage in the Italian three-tier restrictions: a multilevel approach

## Authors

Luca Dei Bardi^1-2^, Anna Acampora^1^, Laura Cacciani^1^, Mirko Di Martino^1^, Nera Agabiti^1^, Marina Davoli^1^, Giulia Cesaroni^1^

^1^ Department of Epidemiology of the Regional Health Service, ASL Roma 1, Rome, Italy.

^2^ Sapienza University of Rome, Rome, Italy.

## Models’ equations

Per every restriction, we defined Rt as:

${Rt}_{ij}= \alpha+\underline{x}_{ij}^{'}\beta+u_{i}+e_{ij}$

Where $i$ indicates the cluster and $j$ the observation. $\alpha$ is the fixed intercept, $\underline{x}_{ij}^{'}$ is the vector of variables observed in cluster $i$ at the $j$-th observation, $\beta$ the vector of fixed effects (common for every cluster and observation), $u_{i}$ the specific random intercept of cluster $i$ assumed to be normally distributed, and $e_{ij}$ the usual error term of linear regressions assumed to be normally distributed as well. Specifically, we run four different models per tier, as reported in Table 1 in the manuscript. Models were defined as follows.

**Model A) in Table 1:**

$${Rt}_{ij}= \alpha+{days}_{ij}\beta+u_{i}+e_{ij}$$

${days}_{ij}$ is the cardinal number of days (0, 1, 2, …), since the models are stratified per each restriction, $\beta$ indicates the effect of passing days in each tier. That is the overall effect of the restriction.

**Model B) in Table 1:**

$${Rt}_{ij}= \alpha+{PED}_{i}\beta+u_{i}+e_{ij}$$

${PED}_{i}$ is the average effect of the province’s economic disadvantage over the Rt.

**Model C) in Table 1:**

$${Rt}_{ij}= \alpha+{days}_{ij}\beta_{days}+{PED}_{i}\beta_{PED}+{days}_{ij}\cdot{PED}_{i} \beta_{int}+u_{i}+e_{ij}$$

${days}_{ij}\cdot{PED}_{i}$ indicates the interaction term between the number of days into the restriction and the province’s economic disadvantage. It is a cross-level interaction because the variable days changes per every cluster $i$ per every observation $j$, while the PED is constant in every observation once the cluster is defined.

**Model D) in Table 1:**

${Rt}_{ij}= \alpha+{days}_{ij}\beta_{days}+{PED}_{i}\beta_{PED}+{days}_{ij}\cdot{PED}_{i} \beta_{int}+{age}_{i}\beta_{age}+{density}_{i}^{ML}\beta_{ML}+ + {density}_{i}^{MH}\beta_{MH}+{density}_{i}^{H}\beta_{H}+{repartition}_{i}^{C}\beta_{C}+{repartition}_{i}^{S}\beta_{S}+u_{i}+e_{ij}$

${age}_{i}$ indicates the share of people aged 0-5. ${density}_{i}^{ML}$, ${density}_{i}^{MH}$, and ${density}_{i}^{H}$ are three dichotomic variables representing, respectively, the Medium-Low, Medium-High, or High population density of the cluster. Their betas are effects compared to provinces with Low population density. ${repartition}_{i}^{C}$ and ${repartition}_{i}^{S}$ are two dichotomic variables that indicate, respectively, the provinces belonging to the Central or Southern geographical repartition. Their betas are effects compared to Northern provinces.

## Supplementary Figures

**Figure S1**: Pandemic risk and restriction levels by Italian regions Nov 2020 - May 2021.
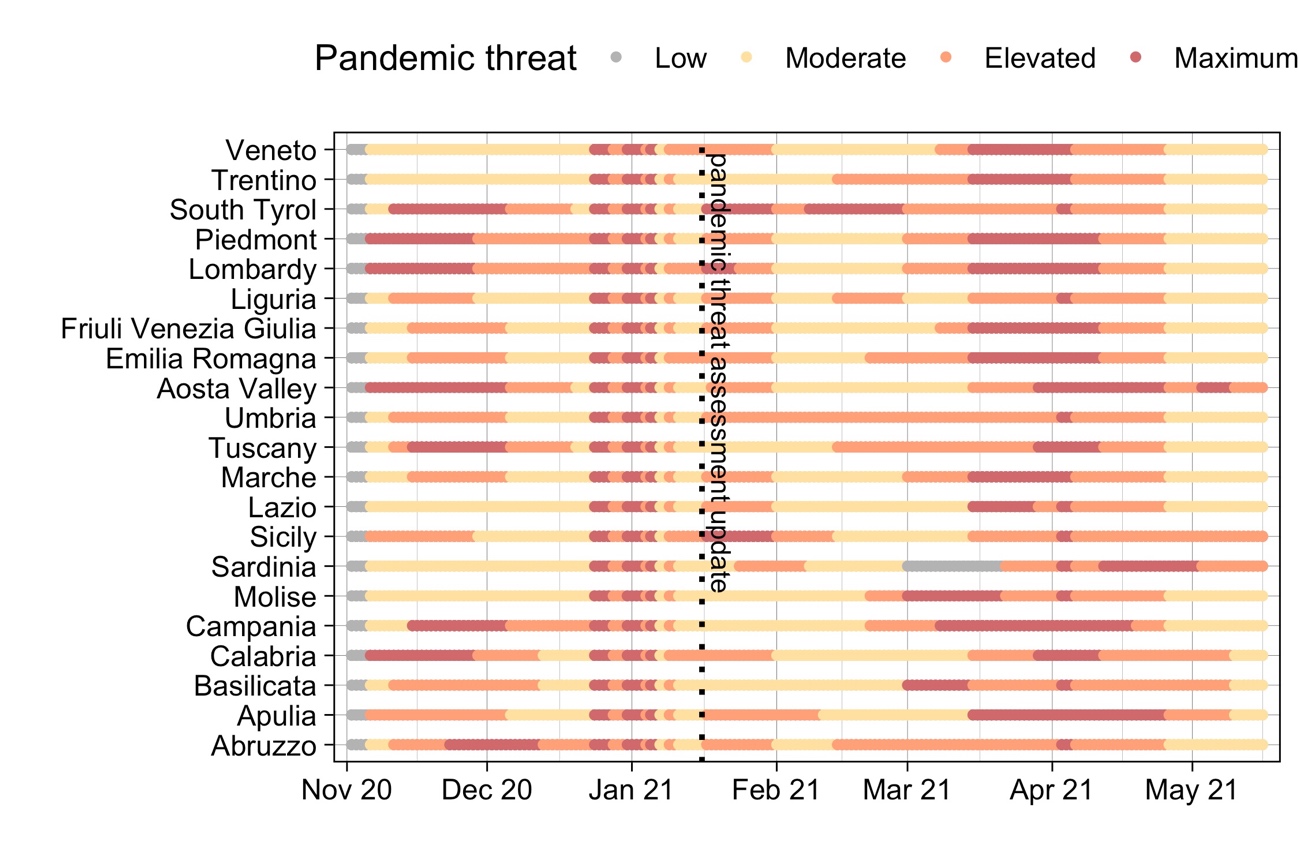


**Figure S2**: SARS-CoV-2 incidence and Cori’s Reproduction number (Rt) in Italy. February 2020 - December 2021.


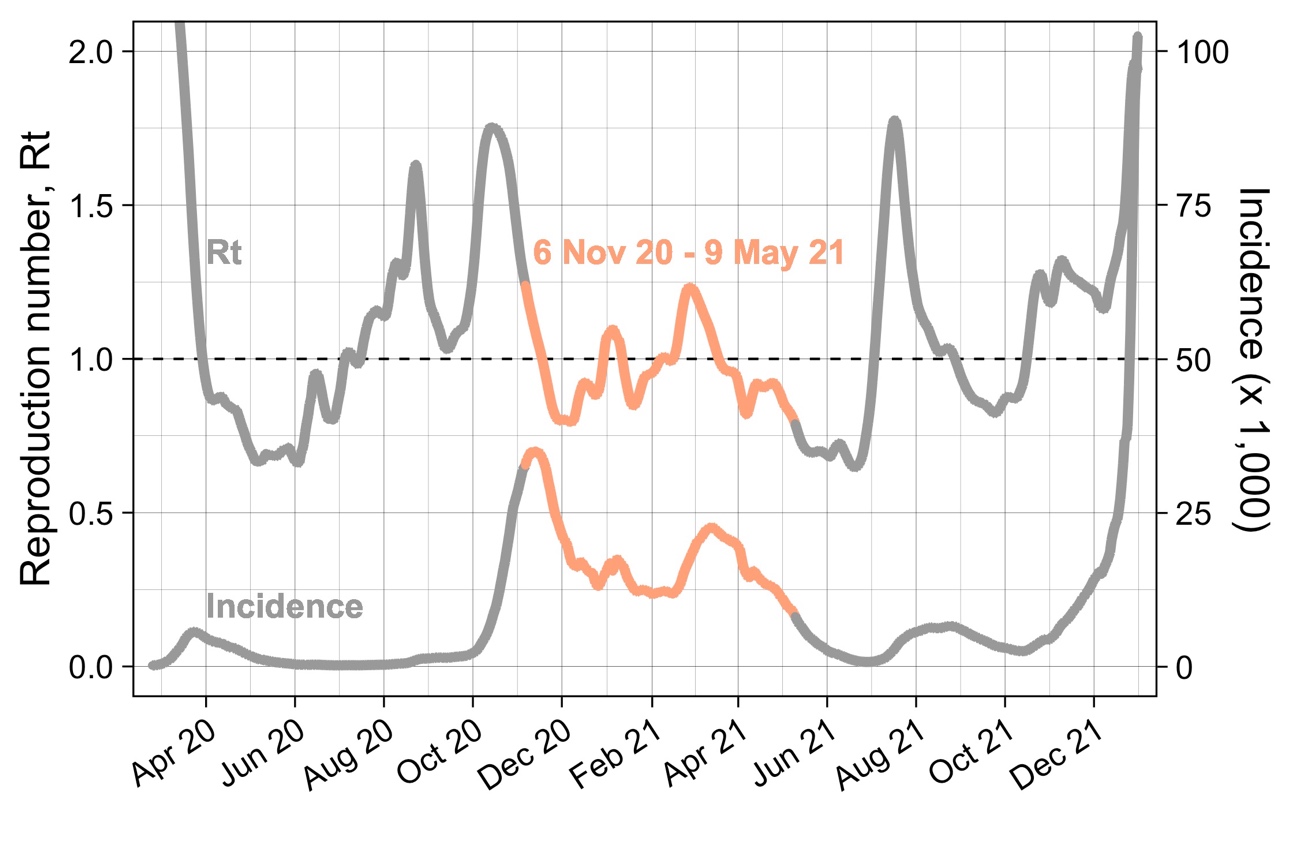


## Supplementary table

**Table S1**: Sensitivity analyses for the association between economic disadvantage and SARS-CoV-2 spread by restriction tier.

|  | **Yellow (ICC = 0.418)** | | | **Orange (ICC = 0.632)** | | | **Red (ICC = 0.553)** | | |
| --- | --- | --- | --- | --- | --- | --- | --- | --- | --- |
|  | **Est.** | **SE** | **p** | **Est.** | **SE** | **p** | **Est.** | **SE** | **p** |
| **1) restrictions >= 7 days** | | |  |  |  |  |  |  |  |
| Intercept | 0.883 | 0.028 | < 0.01 | 0.944 | 0.035 | < 0.01 | 1.091 | 0.040 | < 0.01 |
| days | 0.006 | 5E-04 | < 0.01 | 0.001 | 4E-04 | < 0.01 | - 0.018 | 0.001 | < 0.01 |
| SED | - 0.003 | 0.003 | 0.27 | 0.006 | 0.003 | 0.07 | 0.013 | 0.004 | < 0.01 |
| SED x days | -1E-04 | 3E-05 | < 0.01 | -6E-04 | 3E-05 | < 0.01 | 4E-04 | 4E-05 | < 0.01 |
| **2) weighted estimates** | | |  |  |  |  |  |  |  |
| Intercept | 0.933 | 0.027 | < 0.01 | 0.973 | 0.026 | < 0.01 | 0.993 | 0.030 | < 0.01 |
| days | 0.004 | 4E-04 | < 0.01 | 0.002 | 3E-04 | < 0.01 | - 0.017 | 4E-04 | < 0.01 |
| SED | - 0.003 | 0.002 | 0.29 | 0.005 | 0.002 | 0.04 | 0.008 | 0.003 | < 0.01 |
| SED x days | -6E-05 | 3E-05 | 0.04 | -6E-04 | 3E-05 | < 0.01 | 5E-04 | 3E-05 | < 0.01 |
